# The effect of adding dexamethasone to adductor block and IPACK block on acute postoperative, rebound, and chronic pain following knee arthroplasty—1-year follow-up

**DOI:** 10.3389/fmed.2025.1570795

**Published:** 2025-04-29

**Authors:** Svetlana Sreckovic, Darko Milovanovic, Petar Vukman, Marija Djukanovic, Marko Simic, Jovan Vesic, Marija Milenkovic, Milica Aleksic, Nebojsa Ladjevic

**Affiliations:** ^1^Centre of Anesthesia and Resuscitation, University Clinical Center of Serbia, Belgrade, Serbia; ^2^Clinic for Orthopedics Surgery and Traumatology, University Clinical Center of Serbia, Belgrade, Serbia; ^3^Medical School, University of Belgrade, Belgrade, Serbia

**Keywords:** dexamethasone, adductor block, knee arthroplasty, IPACK block, rebound pain, chronic-postsurgical pain

## Abstract

The analgesic efficacy of nerve blocks depends on the duration of local anesthetics, whose effects can be extended with adjuvant drugs. This prospective interventional study aimed to assess the impact of adding dexamethasone to levobupivacaine on the postoperative analgesic efficacy of the adductor canal block (ACB) and IPACK block after knee arthroplasty (TKA), as well as the incidence of rebound and chronic postsurgical pain. One year after TKA, 80 patients were analyzed (dexamethasone vs. control group). Opioid analgesics were administered to 10% of patients in the dexamethasone group and 50% of patients in the control group (*χ*^2^ = 13.393, *p* < 0.001), with no difference in opioid dosage (*p* = 0.368) during the first 24 h postoperatively. Rebound pain was observed in 5% of patients in the dexamethasone group and 30% in the control group (*χ*^2^ = 7.013, *p* = 0.008). Chronic postsurgical pain 1 year after TKA was found in 5% of patients in the control group, without significant differences between the groups. Adding dexamethasone to the local anesthetic for ACB and IPACK blocks, along with a non-opioid scheduling strategy, enhances postoperative pain management, reduces opioid consumption, and helps decrease the occurrence of rebound pain and chronic postsurgical pain 1 year after TKA.

## Introduction

Total knee arthroplasty (TKA) that is followed by inadequately treated postoperative pain can reduce the benefits of early rehabilitation (1). Different types of nerve blocks have been used as part of a multimodal regime for postoperative analgesia after this type of surgery (2–4). The adductor canal block (ACB) and infiltration in the space between the popliteal artery and the capsule of the posterior knee (IPACK block) were confirmed to be an efficient combination in postoperative pain control, reducing the incidence of chronic post-surgical pain after TKA (5–9). However, the analgesic efficacy of nerve blocks, among others, depends on the duration of action of local anesthetics. Adjuvant drugs such as adrenaline, bicarbonates, dexmedetomidine, and dexamethasone are all used to prolong the effects of local anesthetics (10–12). The intravenous dosage of dexamethasone administered as an adjuvant is higher than that given perineurally as an adjunct to local anesthetics in peripheral nerve blocks. Currently, no recommendations exist regarding the dosage of dexamethasone or the type of nerve block (13–16). Approximately 50% of patients may experience rebound pain after the resolution of a nerve block. Rebound pain is characterized by a transient acute increase in postoperative pain once a peripheral nerve block wears off. It typically manifests within 24 h after the nerve block and can result in sleep disturbances, hinder further rehabilitation, and increase opioid consumption (17). This phenomenon is more prevalent in young individuals, those undergoing bone surgery, and in situations where perioperative dexamethasone is not administered (18). Various strategies, including early identification of at-risk patients, educating patients about multimodal analgesia, administering adjuvant medications, or prescribing opioids, can help address rebound pain, although their effectiveness may vary (17, 18).

The study aims to assess the effect of adding dexamethasone to ACB and IPACK blocks on acute postsurgical pain scores, the occurrence of rebound pain, and chronic pain in these patients after 1 year of TKA.

## Materials and methods

### Patients and study design

**Figure 1:**
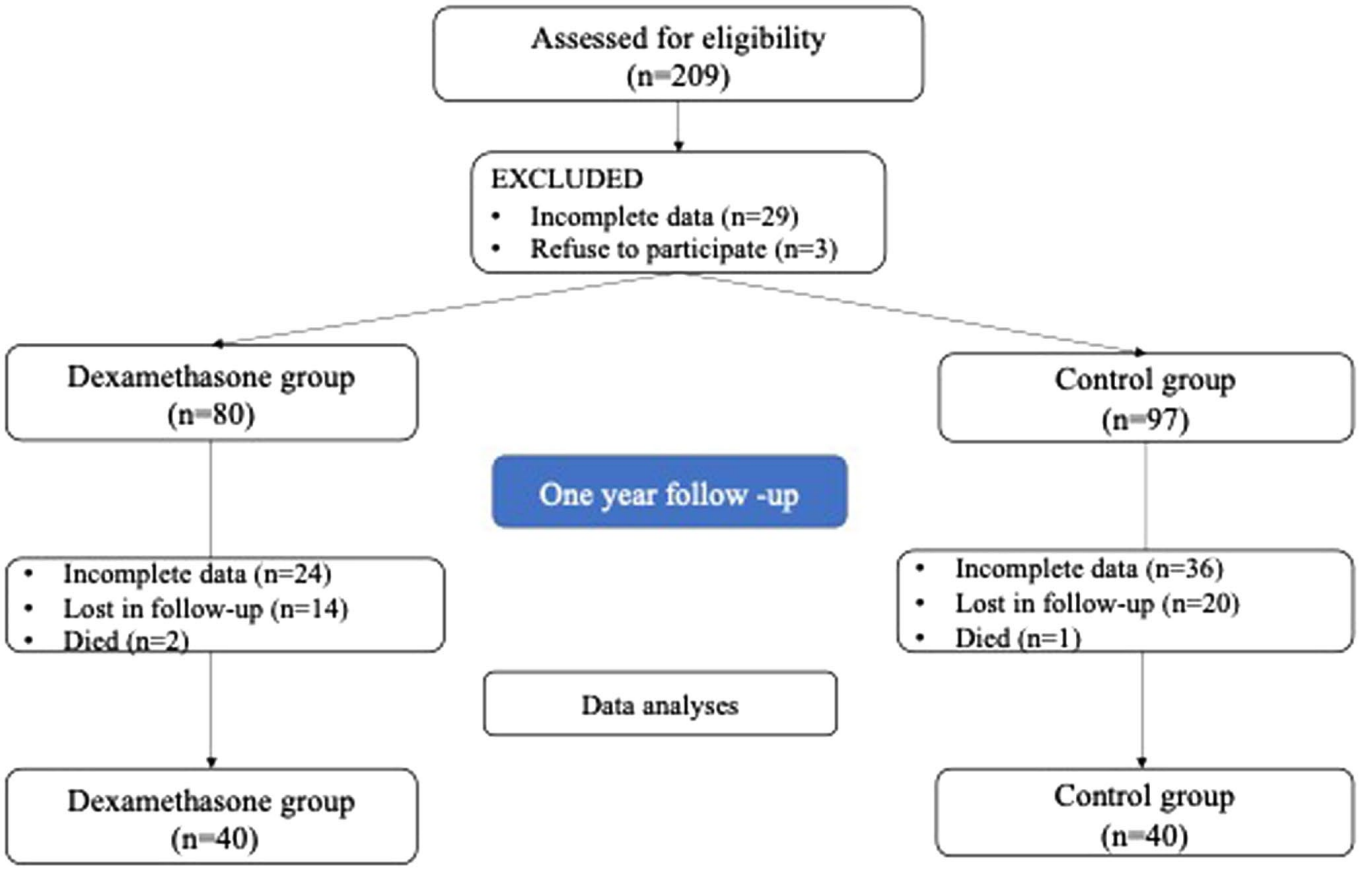
Patient selection and study flow.

This prospective interventional study included patients who underwent elective TKA after receiving approval from the Ethics Committee (No 622/5 24, January 2022). The study was conducted from February 2022 to July 2023 at the Clinic for Orthopedic Surgery and Traumatology, University Clinical Centre of Serbia, by the principles of the Helsinki Declaration. Written informed consent was obtained from all study participants (Figure 1).

Inclusion criteria included patients aged 40–90 years, with an ASA physical status of I–III, receiving the same type of implant, and experiencing pain solely in the knee for surgery (without pain in any other body part). Exclusion criteria comprised individuals with incomplete medical records, refusal of spinal anesthesia or regional block, contraindications to a nerve block, allergies to local anesthesia, opioid use within 30 days prior to surgery, and conditions (such as drug or alcohol abuse, and mental illness) that could compromise rehabilitation.

### Intervention

ACB and IPACK blocks were administered by an experienced anesthesiologist familiar with this technique at the end of surgery. During the block procedure, vital parameters such as electrocardiography, non-invasive blood pressure, and oxygen saturation were monitored, and non-opioids were initiated. In one group of patients, dexamethasone (4 mg for ACB and 4 mg for IPACK blocks) was added to the local anesthetic.

TKA was completed in a bloodless field using a tourniquet inflated to 300 mmHg, without resurfacing the patella, and under spinal anesthesia. Spinal anesthesia was performed with the patients seated. At the L3–L4 level, 3 mL of local anesthetic (0.5% levobupivacaine) was injected.

ACB was performed at the adductor canal’s midpoint after identifying the sartorius muscle using the linear probe (10–12 MHz). Approximately 15 mL of 0.33% levobupivacaine was injected laterally into the femoral artery.

The IPACK block was performed using a curved (2–5 MHz) transducer positioned 2–3 cm above the patella on the medial thigh, flexed at the knee. The needle was inserted into the space between the popliteal artery and the femur, and 15 mL of 0.33% levobupivacaine was injected.

Paracetamol 1 g IV and 30 mg of ketorolac IV were used as non-opioid medications. They were administered alternately, starting with one and introducing the other in the third hour (ketorolac/paracetamol every 6 h).

In the first 48 h following surgery, pain intensity was assessed using the Numerical Rating Scale (NRS, where 0 indicates *no pain* and 10 signifies *the worst pain possible*) at several intervals: 1, 2, 3, 4, 6, 8, 12, 16, 20, 24, 28, 32, 36, 40, 44, and 48 h.

If the pain score was four or higher on the NRS scale, morphine was administered intravenously at a dose of 1 mg every 10 min until the pain intensity decreased. A standardized morphine milligram equivalent (MME) was used to express opioid consumption.

Patients reported postoperative complications, including nausea and drowsiness. Rebound pain, wound drainage, and urinary tract infections were also noted. Rebound pain was defined as a pain intensity NRS score exceeding seven following the resolution of the nerve block.

The same patients were assessed 1 year after surgery by pain specialists for chronic post-surgical pain (CPSP), defined as the persistence of pain in the surgical knee according to the International Classification of Diseases, Eleventh Revision (ICD-11) (19).

### Statistical analysis

The primary outcome measure was the Numerical Rating Scale (NRS) score. Based on preliminary experiments involving 10 patients, the mean 24-h NRS scores for the two groups were 2.2 and 1.4, with standard deviations of 0.91 and 0.87, respectively. Following the requirements of a bilateral test, with *α* set at 0.05 and a power of 90% (1−*β*), along with a projected dropout rate of 30%, the required sample size was calculated to be *n* = 38 patients per group. Therefore, this study included 40 patients per group.

Data analysis was conducted in the statistical program R [version 4.3.1 (2023-06-16 ucrt)—“Beagle Scouts”; Copyright (C) 2023 The R Foundation for Statistical Computing; Platform: x86_64-w64-mingw32/x64 (64-bit)] (available at: www.r-project.org; retrieved: 21 August 2023). Numeric data were tested for normal distribution using a normal K–K plot and histogram, as well as Kolmogorov–Smirnov and Shapiro–Wilk. Data were expressed as mean ± standard deviation (SD). All statistical tests were two-sided, and the *p*-value of <0.05 was statistically significant. To test the difference between the examined groups, and depending on the nature of the examined parameters, the Pearson *χ*^2^ test, Fisher’s exact test, and Wilcoxon rank sum test were used.

## Results

The groups did not differ in patient characteristics such as age, gender, BMI, and ASA status. The average age in the dexamethasone group was 67 years, while in the control group, it was 70 years. Seventy percent of the dexamethasone group were male, compared to 55% in the control group; however, this difference was not statistically significant (*χ*^2^ = 1.92, *p* = 0.166) (Table 1). Normal body weight (BMI = 18.5–24.9 kg/m^2^) was observed in 17.5% of the dexamethasone group and 10% of the control group. Additionally, 47.5% of the dexamethasone group and 65% of the control group were classified as overweight (BMI = 25–29.9 kg/m^2^) (*p* = 0.46). Most patients in both groups had an ASA status of II (55% vs. 52.5%), with no significant difference between them (*χ*^2^ = 0.028, *p* = 0.986) (Table 1).

**Table 1:** Patient characteristics.

Characteristics	Dexamethasone group (*n* = 40)	Control group (*n* = 40)	*P*
Age (y), mean (SD)	66.85 (9.1)	70.5 (6.46)	0.109
TV (m), mean (SD)	1.69 (0.09)	1.7 (0.09)	0.396
TT (kg), mean (SD)	81.2 (12.67)	83.98 (9.75)	0.194
BMI (kg/m^2^)	28.22 (3.36)	28.62 (2.97)	0.4009
Normal weight, *n* (%)	7 (17.5)	4 (10)	0.46
Overweight, *n* (%)	19 (47.5)	26 (65)	
Obese, *n* (%)	14 (35)	10 (25)	
Sex, *n* (%)			0.166
Male	28 (70)	22 (55)	
Female	12 (30)	18 (45)	
ASA physical status, *n* (%)			0.986
ASA I	0 (0)	1 (2.5)	
ASA II	22 (55)	21 (52.5)	
ASA III	18 (45)	18 (45)	

BMI, body mass index; ASA, American Society of Anesthesiologists.

In the first 24 h, 97.5% of patients in both groups experienced mild pain intensity (NRS < 3), which was statistically significantly higher in the control group (1.08 ± 0.27 vs. 1.62 ± 0.63) (*p* < 0.001). There was also a statistically significant difference between groups regarding the number of patients at various time points during the first 24 h postoperatively (Table 2). While performing activities, 85% of patients in the dexamethasone group had an average pain intensity of 1.41 ± 0.61. In comparison, 67.5% of patients in the control group experienced pain but with a significantly higher average intensity of 1.74 ± 0.53 (*p* = 0.016) (Table 2).

**Table 2:** Pain score in the first 24 h postoperatively.

Pain after surgery, at rest	In pain—*n* (%)			Pain (NRS)—mean (SD)		
	Dexamethasone (*n* = 40)	Control group (*n* = 40)	*p*	Dexamethasone (*n* = 40)	Control group (*n* = 40)	*p*
1 h	8 (20)	1 (2.5)	0.03*	1 (0)	1 (0)	–
2 h	12 (30)	26 (65)	0.002*	1.08 (0.29)	1.54 (0.71)	0.04*
3 h	17 (42.5)	34 (85)	<0.001	1.53 (0.8)	1.65 (0.92)	0.706
4 h	30 (75)	39 (97.5)	0.003*	1.2 (0.48)	1.92 (1.48)	0.007*
6 h	35 (87.5)	40 (100)	0.06	1.4 (0.69)	2.42 (1.8)	0.0013*
8 h	36 (90)	38 (95)	0.67	1.42 (0.6)	2.58 (2)	0.0017*
12 h	34 (85)	37 (92.5)	0.48	1.85 (1.05)	2.22 (1.25)	0.129
16 h	37 (92.5)	37 (92.5)	0.67	2 (1.47)	2.65 (1.81)	0.031*
20 h	37 (92.5)	35 (87.5)	0.71	1.65 (0.72)	2.11 (1.13)	0.045*
24 h	37 (92.5)	36 (90)	0.69	1.68 (0.63)	1.89 (0.85)	0.367
Within 24 h	39 (97.5)	39 (97.5)	0.47	1.08 (0.27)	1.62 (0.63)	<0.001
Pain during activity	34 (85)	27 (67.5)	0.07	1.41 (0.61)	1.74 (0.53)	0.016*

*p < 0.05.

In the dexamethasone group, 92.5% of patients experienced pain 24–48 h after surgery, with an average intensity of 1.38 ± 0.55. This was not significantly different from the control group, where 87.5% reported pain at an intensity of 1.31 ± 0.53 (Table 3). At 48-h post-surgery, the incidence and intensity of pain were comparable between the groups (Table 4).

**Table 3:** Postoperative opioid consumption.

Opioids consumption	Patients who needed opioids—*N* (%)			Dose of opioids (mg)—mean (SD)		
	Dexamethasone (*n* = 40)	Control group (*n* = 40)	*p*	Dexamethasone (*n* = 40)	Control group (*n* = 40)	*p*
Within 24 h	4 (10)	20 (50)	**<0.001**	5 (0)	6 (2.05)	0.368
24–48 h	0 (0)	2 (5)	0.47	0 (0)	5 (0)	–
Total	40 (100)	40 (100)		40 (100)	40 (100)	

*p < 0.001.*

**Table 4:** Pain intensity 28–48 h postoperatively.

Time	In pain—*n* (%)			Pain (NRS)—mean (SD)		
	Dexamethasone (*n* = 40)	Control group (*n* = 40)	*p*	Dexamethasone (*n* = 40)	Control group (*n* = 40)	*p*
28 h	35 (87.5)	34 (85)	0.75	1.63 (0.65)	1.62 (0.78)	0.72
32 h	36 (90)	32 (80)	0.35	1.61 (0.8)	1.69 (1.12)	0.89
36 h	35 (87.5)	31 (77.5)	0.24	1.69 (0.83)	1.45 (0.68)	0.25
40 h	34 (85)	29 (72.5)	0.172	1.35 (0.54)	1.45 (0.57)	0.476
44 h	32 (80)	28 (70)	0.302	1.38 (0.55)	1.21 (0.42)	0.252
48 h	28 (70)	29 (72.5)	0.8	1.21 (0.42)	1.1 (0.31)	0.26
24–48 h	37 (92.5)	35 (87.5)	0.71	1.38 (0.55)	1.31 (0.53)	0.574

**Figure 2:**
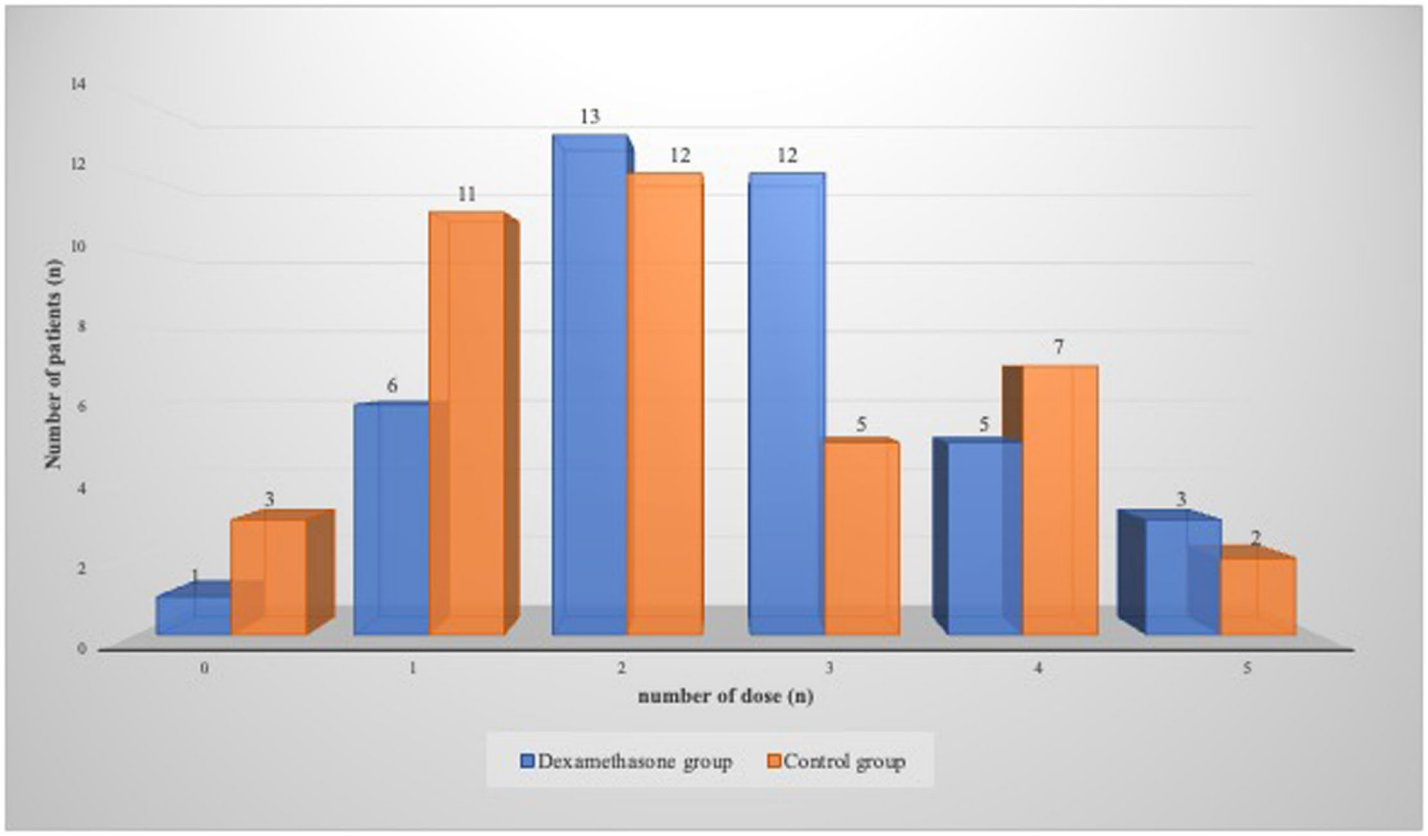
Consumption of non-opioid analgesics 24-48 hours postoperatively.

On the first postoperative day, there was no statistically significant difference between the groups in the number of doses of non-opioid analgesics (*χ*^2^ = 3.392, *p* = 0.64) (Figure 2). Most of the patients in the dexamethasone group were administered two doses, with 13 (32.5%) patients needing this amount, while 12 (30%) patients received three doses of non-opioid analgesics. In the control group, most patients were administered two doses, 12 (30%), one dose, 11 (27.5%), and three doses, 7 (17.5%) of non-opioids (Figure 2).

In the first 24 h after surgery, more patients in the control group requested rescue opioid administration compared to the dexamethasone group (*χ*^2^ = 13.393, *p* < 0.001). However, no statistically significant difference was found in the average dose of opioid analgesics between the two groups. On the first postoperative day, only two patients in the control group received 5 mg of opioids (Table 3).

Rebound pain (*χ*^2^ = 7.013, *p* = 0.008), nausea (*χ*^2^ = 4.505, *p* = 0.034), and drowsiness (*χ*^2^ = 12.655, *p*<0.001) were statistically more frequent in the control group (Table 5). Two patients had urinary tract infections in each group (Table 5). One year after TKA, CPSP was present in 5% of patients in the group without dexamethasone (Table 5).

**Table 5:** Postoperative complications.

Characteristics	Dexamethasone group (*n* = 40)	Control group (*n* = 40)	*p*-value
Postoperative complications			
Rebound pain, *n* (%)	2 (5%)	12 (30%)	0.008*
Nausea, *n* (%)	0 (0%)	6 (15%)	0.034*
Drowsiness, *n* (%)	3 (7.5%)	18 (45%)	<0.001
Wound drainage, *n* (%)	0 (0%)	0 (0%)	–
Urinary tract infection, *n* (%)	2 (5%)	2 (5%)	0.61
Characteristics, 1 year after surgery			
Chronic post-surgical pain, *n* (%)	0 (0%)	2 (5%)	0.47

*p < 0.05.

## Discussion

Our study’s findings indicated that adding dexamethasone to the local anesthetic for ACB and IPACK blocks, along with a non-opioid scheduling strategy, improves postoperative pain management during the first 24 h following TKA. Patients reported mild pain intensity in both groups, with differences observed in the number of patients experiencing pain and in pain intensity. Furthermore, fewer patients in the dexamethasone group required opioids and reported a lower incidence of nausea and drowsiness. Dexamethasone also reduced the occurrence of rebound pain and chronic postsurgical pain 1 year after TKA.

The implantation of a total knee prosthesis aims to reduce pain, minimize disability, and improve quality of life (20). The choice of anesthesia for TKA, whether general or neuraxial, should be customized based on the individual patient’s preferences, considering comorbidities and their level of optimization (21, 22). Neuraxial anesthesia is frequently preferred for these patients, as it reduces the risk of acute renal and respiratory failure, thromboembolic complications, the need for blood transfusions, and infections. This type of anesthesia has also been associated with decreased admission rates to the intensive care unit, reduced re-admission rates, and shorter hospital stays (3, 23). In our study, all patients received spinal anesthesia, and 54% were classified as ASA status II.

TKA is associated with high-intensity pain, not only in the first 24 h postoperatively. Additionally, early rehabilitation, which is crucial for these patients, complicates the selection of an appropriate analgesic modality. The optimal approach to postoperative pain management should be as selective as possible, focusing on the area near the surgical site to provide adequate analgesia without inducing muscle weakness, thereby facilitating early rehabilitation (24, 25). Combining the ACB and IPACK blocks may provide a potential therapeutic solution after TKA (26). Guo et al. included 14 studies in their meta-analysis, demonstrating that adding the IPACK block to the ACB is an effective method for pain management. This combination reduces postoperative VAS scores, decreases cumulative morphine use, shortens hospital stays, and enhances patients’ activity levels after TKA without causing additional side effects (26). These findings were supported by Wang et al. in a meta-analysis comparing the addition of the IPACK block to the ACB within a multimodal pain management protocol, showing that this combination reduces opioid consumption in the early postoperative period (27). However, despite the numerous advantages and technical improvements using ultrasound, nerve blocks are limited by the duration of LA (28). An effective alternative is to place a nerve catheter to provide a continuous block; however, the costs, complexity of placement, and potential morbidity from catheter infections and accidental dislodgement outweigh its benefits (3, 28). Therefore, administering a single dose of an adjuvant to extend the duration of LA is preferable (13, 15, 28). Dexamethasone can be added to local anesthesia to prolong its effects; however, the method of administration—whether perineural or systemic—remains a topic of debate (11, 29). Baeriswyl et al. conducted a meta-analysis comparing the analgesic efficacy of both methods, which included 11 randomized studies with 914 patients. The duration of analgesia was significantly longer with perineurally administered dexamethasone compared to systemic administration, showing an average difference of 3 h. The perineural administration of dexamethasone, in combination with bupivacaine but not with ropivacaine, slightly extended the duration of analgesia without affecting other pain-related outcomes when compared to systemic dexamethasone (30). Another systematic review indicated that both perineural and intravenous administration of dexamethasone effectively prolongs sensory block duration and reduces postoperative pain intensity and opioid consumption compared to placebo. When comparing intravenous and perineural dexamethasone, perineural administration was found to be more effective than intravenous. The sensory block lasted over 3 h, and postoperative pain intensity was significantly lower in the perineural dexamethasone group (13). However, an optimal dose of perineurally administered dexamethasone has not been defined. Kirkham et al., in the meta-analysis, showed that the dose of 4 mg dexamethasone in combination with short/intermediate and long-acting local anesthetics prolonged analgesia after brachial plexus blockade in patients undergoing upper extremity surgery (31). Moreover, the potential neurotoxic effects of dexamethasone, including bioenergetic disruption, mitochondrial dysfunction, oxidative stress, and apoptosis, should be considered (32).

Dexmedetomidine, another adjuvant, is combined with dexamethasone to prolong the duration of the blockade. Maagaard et al. demonstrated in their meta-analysis that this combination increased the duration of analgesia compared to placebo and dexmedetomidine alone. This combination likely provided an analgesia duration comparable to dexamethasone concluding that using dexamethasone as the sole adjunct is reasonable if the goal is to enhance analgesia duration (16). Dexamethasone significantly lowered the need for opioids by prolonging the analgesic effect of LA and reducing rebound pain (33, 34). Singh et al. have shown in a meta-analysis that dexamethasone administered either intravenously or perineurally decreases the incidence of rebound pain after a peripheral nerve block in postoperative analgesia (17). In our study, 10% of patients in the dexamethasone group received an opioid analgesic at a dose of 5 mg, whereas 50% of patients in the control group received a dose of 6 mg.

Up to 44% of patients experienced chronic pain after TKA, with 15% suffering from severe pain. This condition affects quality of life, leads to dissatisfaction, and increases the likelihood of revision surgery (35). Dexamethasone, administered in one or two intravenous doses of 24 mg, did not influence the development of chronic pain or physical function 3 years after TKA (36). However, the combination of ACB and IPACK blocks reduces the incidence of chronic pain and enhances functional tests 2 years after TKA (9).

## Conclusion

Adding dexamethasone to the local anesthetic for ACB and IPACK blocks, along with a non-opioid scheduling strategy, improves postoperative pain management after TKA. It also decreases opioid consumption, nausea, and drowsiness. Furthermore, dexamethasone contributes to reducing the occurrence of rebound pain and chronic postsurgical pain 1 year following TKA.

## Data Availability

The raw data supporting the conclusions of this article will be made available by the authors, without undue reservation.
